# Is a single editorial sufficient?

**DOI:** 10.4103/0019-5545.44911

**Published:** 2009

**Authors:** Nitin Gupta

**Affiliations:** Consultant Psychiatrist-South Staffordshire and Shropshire Healthcare NHS Foundation Trust and Honorary Senior Lecturer-Faculty of Health, Staffordshire University, United Kingdom. E-mail: nitingupta659@yahoo.co.in

Sir,

It was extremely heartening to see that the most recent issue of the Indian Journal of Psychiatry carried an Editorial on Sexual Medicine.[1] What was further interesting to note was that the Editorial being an outcome of the joint efforts of our Esteemed Editor-in-Chief (TSS) and a Senior Psychiatrist who has specialised in the practice of Psychosexual Medicine for over 2 decades (AA). The authors provide a brief snapshot of the current body of research and highlight pertinent issues contributing to the lack of growth of Sexual Medicine in India. Pertinent directions for the future are suggested to further the science of sexual medicine.

Considering that the research in sexual medicine in India had started way back in the 1980s,[1] in my opinion, the contribution so far is quite dismal. To rectify this situation, I would like to add some suggestions to those provided in the editorial. These could be seen at two levels. Firstly, we need to recognise and utilise one of the major strengths of health service delivery in India: community-based care. Collaborative inputs from the specialty of community medicine and other concerned speciality(ies) have led to high-quality consistent work surrounding epidemiological surveys and delivery of healthcare at the primary level (e.g. various national programs). Such opportunities can be generated and models replicated by psychiatrists at local, state, and national level. Hence, it may be worthwhile to open up a meaningful dialogue and develop strong and consistent collaborative links with community medicine and the primary care services. This shall help in providing appropriate sex education and in addressing the difficulties in attitudes and knowledge and treatment seeking pathways of patients with various sexual disorders. Also, through this collaborative approach we can utilise the expertise that the speciality of community medicine has developed over the years in the fields of epidemiology and healthcare delivery. For the clinic-based approach the speciality to consider along similar lines is Obstetrics and Gynaecology; this will be especially pertinent for women suffering with sexual dysfunction. This is consistent with the editorial's reference to the community-based studies and integration of various specialities.[1]

Secondly, we must recognise that the positive initiatives taken up by the Indian Psychiatric Society (IPS) in recent years[1] run the danger of getting lost in the myriad of time if not persisted with. Practice Guidelines and CME are helpful, but there is still the ongoing need for consistent standards of implementation and evaluation of the guidelines and holding more of these educational events. IPS should consider ways of highlighting the need for and developing the speciality of Sexual Medicine. Suggested options that could be considered are: a special issue of our journal devoted to various facets of sexual medicine, regular symposia/sessions in the Annual Conference (maybe, even a Thematic symposium by an IPS President in the near future), a speciality section of IPS, and developing formal links with relevant specialities (urology, neurology, obstetrics and gynaecology, community medicine) and considering participation in their Annual scientific conferences. The editorial emphasises the need for the psychiatrists to take on more responsibility and act as a co-ordinators. It is up to us as members and office bearers of the IPS to give serious thought to the development of this very important speciality. The IPS needs to put in a concerted effort towards formulating a long-term agenda to serve the public in this area (by recognising and alleviating the prevailing psychosexual illnesses).

I am reminded of the famous words of Neil Armstrong- “*That's one small step for man; one giant leap for mankind*”.[2] The editorial's contents reflect this quote's concept and pathos, and I hope that this will be followed through by the IPS.
